# Coverage Area Decision Model by Using Unmanned Aerial Vehicles Base Stations for Ad Hoc Networks

**DOI:** 10.3390/s22166130

**Published:** 2022-08-16

**Authors:** Saqib Majeed, Adnan Sohail, Kashif Naseer Qureshi, Saleem Iqbal, Ibrahim Tariq Javed, Noel Crespi, Wamda Nagmeldin, Abdelzahir Abdelmaboud

**Affiliations:** 1 Department of Computing and Technology, Iqra University, Islamabad 44000, Pakistan; 2 University Institute of Information Technology, PMAS-Arid Agriculture University, Rawalpindi 46000, Pakistan; 3 Center of Excellence in Artificial Intelligence (CoE-AI), Department of Computer Science, Bahria University, Islamabad 44000, Pakistan; 4 Department of Computer Science, Allama Iqbal Open University, Islamabad 44000, Pakistan; 5 Institut Polytechnique de Paris Telecom SudParis Evry, 91000 Evry, France; 6 Department of Information Systems, College of Computer Engineering and Sciences, Prince Sattam bin Abdulaziz University, Al-Kharj 11942, Saudi Arabia; 7 Department of Information Systems, College of Science and Arts, King Khalid University, Muhayil Asir 61913, Saudi Arabia

**Keywords:** UAV, networks, coverage, base station, mobility, delay

## Abstract

Unmanned Aerial Vehicle (UAV) deployment and placement are largely dependent upon the available energy, feasible scenario, and secure network. The feasible placement of UAV nodes to cover the cellular networks need optimal altitude. The under or over-estimation of nodes’ air timing leads to of resource waste or inefficiency of the mission. Multiple factors influence the estimation of air timing, but the majority of the literature concentrates only on flying time. Some other factors also degrade network performance, such as unauthorized access to UAV nodes. In this paper, the UAV coverage issue is considered, and a Coverage Area Decision Model for UAV-BS is proposed. The proposed solution is designed for cellular network coverage by using UAV nodes that are controlled and managed for reallocation, which will be able to change position per requirements. The proposed solution is evaluated and tested in simulation in terms of its performance. The proposed solution achieved better results in terms of placement in the network. The simulation results indicated high performance in terms of high packet delivery, less delay, less overhead, and better malicious node detection.

## 1. Introduction

One of the fundamental requirements of any new and advanced network, especially digital agriculture networks where full-service coverage needed. Precision agriculture optimizes the agricultural processes to certify final production. The users in these networks are always looking for full coverage and strong signal networks and services. In digital agriculture networks, the services and coverage should be available anytime and anywhere. The choice of users is always based on these basic criteria, especially in cellular networks. With new and integrated technologies, user demand has increased to the point where they need faster, more reliable, and more secure data communication [1,2]. This user expectation is fourfold, especially in complex areas based on network systems, such as in a disaster-affected area where the connectivity and permanent infrastructure such as Base Station (BS) deployment is not possible or is difficult. On the other hand, some examples are those where the services and BS are available, but the user density is high. Still, various strategies have been adopted and designed to address these issues and meet user expectations [3,4]. For long-range data communications, high-power transmitters have been adopted, but such communication systems require energy resources. 

In cellular networks, the land areas are distributed into smaller areas called cells. These cellular network cells are joined together to increase the geographical area. Normally, each of these cells is served by a fixed location tower known as a Base Transceiver Station (BTS) or Base Station (BS) [5]. In these networks, the devices are connected with cellular towers (PECO cells/microcells), and smaller cells are connected with large cells, which are further connected with core networks [6]. The smaller cells are further connected directly to the core networks to acquire basic services for users. The cell size is different and considered by using the user’s density in the networks. These cells can be deployed in macro, micro, or pico levels [7,8]. A macrocell usually covers a wide area of radius, which is around 25 km depending upon the density of users. The micro cells normally have a coverage area of up to 2 km, whereas the pico cells are used to cover the smaller areas such as offices, shopping malls, and buildings [9]. The need for a smaller cell size is not only dependent on the coverage area but also affects user capacity. By dividing the cell into smaller levels, the reusability of the channel increases by many folds. The fixed BS provides best-effort connectivity services. However, the BS works in ideal situations such as proper tower foundation space, feasible installation, and obstacle-free areas. While they have many advantages, BS services also suffer from higher setup costs, maintenance issues, and other cumbersome processes. Moreover, where the deployment of physical infrastructure is difficult or there are more chances of natural disasters, such as floods and earthquakes [10], quick restoration and recovery of uninterrupted connectivity services are not possible. Some other issues also exist, such as power supply issues to the BS, generator cost, land issues, coverage, capacity, and road coverage concerns.

To address these issues, Unmanned Aerial Vehicles (UAVs) based on microcellular solutions have been used to offer temporary ground-based solutions [11,12,13]. Such solutions are also being explored for increasing the coverage in fifth-generation (5G) networks. These aerial BSs are deployed using drones, such as Drone Base Stations (DBSs), and continuously adapt their moving directions to provide higher Quality of Service (QoS) for mobile users on the ground [14,15]. The role of UAVs with all new and fast data communication services is to provide broadcasting and point-to-point communication. These new technologies overcome the existing issues in typical cellular networks. These solutions are feasible with more cost-effective solutions, especially where the cellular networks suffer from shadowing, interference, coverage, and other service issues. Single-tier or multiple-tier drones in cellular networks have a significant impact on the desired communication between UAVs and users on the ground. Most of the researchers used similar power and similar altitude in the case of multiple UAVs, which is not realistic as user densities may vary, and the UAVs need to adjust their altitude based on these densities. Whenever the UAV density increases, the distance among nodes also decreases and causes interference. The coverage area must be maximum, where the user’s probability is covered with a specified threshold and the optimal number of DBS to cover a larger area. Authors in [16] discussed the service scheduling issues in the Internet of drones network. Due to the high mobility of nodes and limited resources in terms of communication range, bandwidth, and limited zone service providers, service scheduling becomes a critical issue, especially for downloading and uploading data. Authors in [17] discussed the security issues in the Internet of drones network and proposed a lightweight and privacy-preserving mutual authentication scheme for session establishment. The existing studies have suffered from computational complexities in deploying security. In another study [18], authors discussed the lightweight digital solution for drones, tackling the man-in-the-middle attack. This protocol uses a digital signature based on command messages by using a chaotic system. Authors in [19] discussed the flooding attacks and presented a solution to handle this attack in drone networks. The lightweight solution is presented where each drone counts the number of packets which are sent within a predefined time interval.

Different solutions have been designed to address these issues in [20]; numerical algorithms are used as an alternative. Some other studies divided the area based on user distribution data, calculating distance, allocation of DBS, cluster formation, establishing the DBS at the optimal location, and coverage area specification in 2D and 3D planes [21,22]. These studies obtain the signals from source nodes because of effective diversions and reflections. In another study [23], the authors considered the probability of having LoS connections between receiver and transmitter. There is a need to design a solution to find the best position of the UAVs-BS in a microcell environment by using better techniques and providing optimal coverage and increased capacity throughout the service area. This paper aims to improve the cellular network services by optimally using the UAVs and DBS placement. To achieve the aim of this research, the following objectives are in line.

- To design a solution for optimal cellular coverage by establishing optimal numbers of BSs in the cellular network area.
- To design a controlling solution to manage and reallocate the nodes as per requirements.
- To evaluate the solution for better resource allocations in the cellular network.

The rest of the paper has the following sections. Section 2 reviews the literature in the field of routing. Section 3 discusses the design and development phases of the proposed solutions. Section 4 illustrates the evaluation steps and results, whereas the last section concludes the paper with future work.

## 2. Related Work

A heuristic technique was suggested by Kalantari et al. [24] as a way to locate a drone BS in any area with a variety of user densities. With a set of users and a quality-of-service target in mind, the suggested solution looks for the fewest drones necessary to cover all of the users (QoS). After that, the drone BSs are eliminated, whose removal has no impact on the network’s quality. Because properly situated drone base stations can serve the greatest number of users, Bor-Yaliniz et al. [25] emphasized the drawbacks of the drone BSs placement challenge. The impact of the environment on the line of sight should also be considered. For this reason, the authors developed a 3D placement challenge to increase the network’s revenue as much as possible. A similar mixed-integer non-linear quadratically constrained optimization problem was also defined by the authors, and they suggest a numerical solution that is computationally effective by applying some mathematical tricks.

Fotouhi et al. [26] emphasized the advantages of a drone’s dynamic repositioning during the service in response to the active and mobile user. They increase the spectral effectiveness of small drone cells. Three algorithms for self-determining dynamic relocation for drone BSs were suggested and tested by the authors. Due to Wi-Fi’s limited throughput and the uncontrolled nature of the unlicensed spectrum in which it works, Deruyck, et al. [8] chose LTE technology over it. LTE femtocell base stations are mounted on the drones. The deployment tool’s algorithm, which the authors investigated, consists of four distinct steps. The development of the deployment tool enables determining the number of drones that are required as well as their best placements to maximize user coverage. The user is connected to the base station with the lowest route loss after the path loss is assessed and generated for the traffic. The first findings of this study indicated that it is promising for an examination of the usage of drones in emergency situations. Based on the scenario and drone assumptions taken into consideration, it was found that the optimal altitude of a drone BS that achieves the necessary coverage with the least transmit power also delivers supreme coverage with two drone BSs in the presence and absence of interference [27].

For a variety of network design factors, including the number of served users or the sum rate of served users for heterogeneous rate requirements in a clustered user distribution, Kalantari et al. [28] suggested a backhaul restricted optimum drone BS placement algorithm. Additionally, the authors looked into the stability of drone BS positioning and the potential impact of user motions on the suggested ideal solution. The lack of dependable wireless backhaul links restricts the deployment of drone base stations (drone BSs). To determine user priorities, two methods are introduced: network-centric and user-centric. These approaches are based on sum rate, pricing differential, signal strength, and content demand. For each approach, a drone BS placement that is 3D backhaul-aware is discovered. In the network-centric and user-centric frameworks, respectively, the total number of serviced users and sum rates are maximized. A macro BS (MBS) and many DBSs that rely on the wireless links to the MBS for backhauling were suggested by Kalantari, et al. [29]. The authors suggested an approach to discover effective 3D placements of DBSs together with user–BS relationships and wireless backhaul bandwidth allocations to optimize the logarithmic sum rate of the users while considering both normal and uRLLC users. Allocating backhaul resources, user association considering user kinds and 3D placement of DBSs are all taken into account simultaneously. A fresh issue formulation that takes fairness into account is offered as well as a numerical solution approach. Using a decomposition method, the user–BS association and bandwidth allocations are determined. A heuristic particle swarm optimization approach is then used to update the DBS sites, determining the user–BS associations while taking user type into account. Allotment of bandwidth for backhaul and access links Khan et al. [30] focused on designing the dynamically positioned 5 G UAV base station algorithms. The main objective of this solution is to capture the spatio-temporal relationship between data demand points and depict the clusters and their centres during well-selected periods. This research simulates the behaviour of the algorithms relying on people’s real routes in downtown Beijing. In a natural disaster in an extremely dense area the placement algorithm can be implemented with a little modification that forms a sample dependent on actual 3GPP minimization of determination tests data and combines the offered algorithms in the perspective of big Internet of Things networks (IoT), optimizing the IoT gateway location equally in terms of battery life (spontaneously, less energy is needed if the IoT gateway is closer) and scalability. In another study, a new mobility model for drone-based stations is proposed, where UAVs can move freely in the network, overlooking the cell limit. To provide a responsible connection to launch backhauling, drones also need a high capability to connect via links with a fixed earthly wireless backhaul centre. Therefore, the operating cost of drones may be high.

Sekander et al. [31] examined the latest technological advancements in drone networks and drone-assisted cellular networks. Then, while numerically demonstrating the ideal intensity and altitude of drones in various tiers, they evaluate the performance of a multi-tier drone network in terms of the spectral efficiency of downlink transmission. The study aims for the optimization of the altitude with power optimization, also dealing with traffic control of signals while keeping the quality optimized for users. The researcher in [32] overcame the complexity of bigger cellular network problems, by dividing the small cells into clusters so that they could be managed easily. Following that strategy, the small cell can be deployed through the drone along with the base station mounted on it. The study of Wang et al. [33] combined two methods, first using the reference signal received power of each ground user to discover hidden mutual interference configurations and then using the K-means clustering algorithm to discover hidden mutual distance structures of the locations of serving users. The authors hypothesized that a temporary wireless network architecture might be efficiently created based on the two learning properties mentioned above.

In [34], the authors presented a real-time adaptive cooperative transmission strategy as an energy-efficient UAV solution. The proposed solution utilized the infrequent battery power during data communication and live operations. The authors considered cooperative relaying communication techniques to minimize energy consumption and enhance the life of batteries operated in terrestrial nodes. To communicate in an energy efficient manner with the Low Altitude Platform (LAP), a source node is analyzed as an option to transmit the data through a direct link to the LAP and relayed links through different possible relay links in the network. This study also emphasized how a source node selects an energy-aware method to communicate with the LAP without negotiating the bit error rate to maintain a minimum level and better QoS. This solution is a hybrid architecture and is specially designed for emergency services. The game–theoretic approach is used for terrestrial terminals on relay selection in multi-LAP aerial–terrestrial systems.

In [35], the authors identified the high traffic demand which leads to lower speed connections and proposed a demand-based network model by using multiple UAVs. In this proposed model, the demand for wireless sensor networks increased rapidly for data collection and provision of more services and the demand for infrastructure-based cellular networks for a more efficient and cost-effective solution. These models used density and cost functions. Due to this model, achieving better capacity, throughput, reliability, and longtime connectivity may be possible. Based on multiple UAVs, deployment models are presented and also provide an algorithm for area mapping (for UAVs). This study adopted a neural-based cost function where the UAV is matched with a particular geographical area. The multiple UAV deployment not only solves the range coverage issue but also offers load balancing and traffic offload solutions. The authors designed a system model by using the macro and small cell user equipment. This model is demand-based and uses density and cost function and then computes the area.

In [36], the authors proposed a drone-as-a-gateway (DaaG) solution by using the mobile gateway and sink system or delay-tolerant system. This solution is based on a drone as a gateway where the actuator is visited by drones, reducing the service delay. The authors also discussed the solution of optimal height for a drone which can be optimized by using multiple algorithms to increase spectral efficiency. This study used three algorithms, Equal Bandwidth Division, Nearest User First, and Least Buffer First, for the dynamic repositioning of the drone BS. In equal bandwidth division, all the active users equally share the resources. In the nearest user first, the nearest user to the BS has the highest level of spectral efficiency. The last buffer drone finds the user with the least remaining data to send and allocates all the resources to that user. The usage of mobile sensing devices with their functionalities is increasing rapidly. However, managing these types of devices in an integrated manner and handling the heterogeneous networks is a huge challenge and problem, especially in wireless sensor and actuator networks. Sensors and actuators are tightly coupled and integrated and not suitable for heterogeneous types of networks. The basic problem in these networks is a combination of heterogeneous networks. In the recent past, there have been many aspects of a drone BS. This solution, such as a dynamic BS, has already been considered and studied by many researchers to improve UAV performance. The problem in dynamic BS allocation is a fixed area which has a minimum transmission range.

In [37], the authors presented a self-healing neural model network and the concept of matrix colouring (in the form of adjacent and isolated cells) for achieving the optimum utilization of UAVs. This study discussed the matrix approach and proper method for the deployment of UAVs as a low energy consumption mechanism. The proposed solution also considered the memory and cost of UAV deployment. The two network techniques are used in this model, including backhaul (unidirectional) and fronthaul (umbrella form). The network model consists of microcells and several BSs, which are capable of maximizing the user equipment (UEs) and connections. The area is subdivided based on two algorithms: (1) for isolated cell identification and (2) for UAV deployment. The proposed approach provides high throughput coverage, optimizes the utilization of UAVs, and also solves the placement problem in a 5G network with low energy consumption and less memory utilization.

In [38], a higher level of an abstraction energy-aware communication protocol based on power management is presented. Power management techniques also included are called power harvesting. The key challenge is the deployment of sustainable batteries to achieve the optimum utilization of energy. The environmental harvesting technique for energy harvesting is trustable, but solar energy supply is a highly time-intensive source. Harvesting components have various protocols for charging characteristics; these characteristics must be matched with each other. A battery is not a good choice for charging, harvesting and storing, or discharging purposes. Solar energy harvesting is implemented to prolong the battery time. All types of energy harvesting, solar, piezoelectric vibration, and thermometric acoustic noise are manageable and also useable. The authors analyzed all types of components relating to solar energy. The existing studies have a common factor that is an area management strategy to evaluate the network performance. In [18], the overall system architecture is also discussed and considers the nodes’ placements for emergency communication. In [39], the authors discussed the software-defined-networks-based (SDN) manageable topology formation to construct a UAV formation. The proposed solution considers the set of graph theories for network evaluation. The authors also used particle swarm optimization (PSO) for the selection process and to maximize the number of interconnected nodes. Table 1 shows the technical comparison of existing solutions.

Different solutions have been designed to address the coverage area issue in cellular networks. Some studies have divided the area based on user distribution data, calculating distance, allocation of DBS, cluster formation, establishing the DBS at the optimal location, and coverage area specification in 2D and 3D planes, which can still obtain the signal from the source due to strong reflections and diversions. The probability of having an LoS connection between a transmitter and a receiver is an important factor for modelling such channels. There is a need to design a solution to find the best position of the UAVs-BS in a microcell environment by using better techniques and providing optimal coverage and increased capacity throughout the services area.

## 3. Proposed UAVs-BS Network Coverage Model

**Figure 1:**
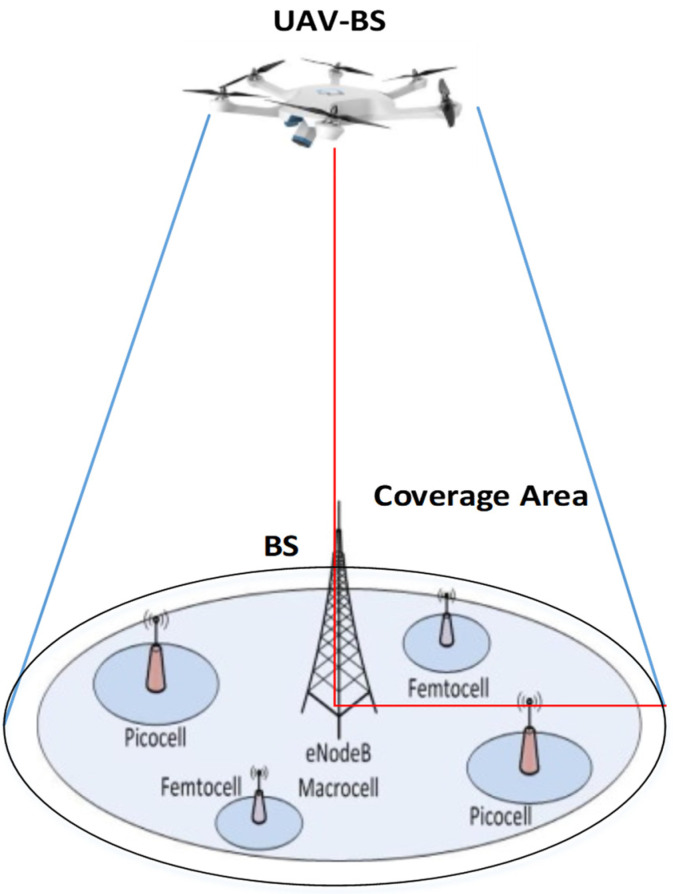
UAV-BS coverage area.

Cellular network coverage is always a challenge due to congested networks and the increased trend of users. Unmanned aerial vehicles (UAVs) in cellular networks have offered a reasonable solution to address the coverage issue. These networks use UAVs as a base station (BS) to improve cellular network coverage. With many positive features, these types of solutions have suffered from some constraints related to installation, optimization with the network, scalability, and link quality issues. Environmental factors have also affected UAV services and performance. The UAVs are controlled and managed for reallocation and position change per requirements. The UAVs have high line-of-sight (LoS), which makes them more realistic for the ground networks. The UAVs-BS is a more feasible solution for ground network users for providing the services and coverage and fulfilling the network requirements. The challenging task of UAVs is finding the optimal position in the microcell. The UAVs-BS is one of the flexible and low-cost solutions to improve the ground network connectivity and provide high data rate services. The coverage area provision is still a challenge, especially in congested networks. Allocation of a UAV-BS needs to be set with maximum and minimum vertical attitude. This section presents a UAVs-BS coverage area model for cellular networks. The complete design and development phases of the proposed model are presented in the next sub-sections. Figure 1 shows a UAV-BS coverage area example where the UAV-BS provides coverage to the cells of the cellular network.

To design the proposed UAVs-BS network coverage model, this paper considered a few assumptions, which are as follows:

- Security is not considered in designing the coverage model.
- The GPS services are available to find the locations and positions of the nodes.
- Mobility and number of nodes are set according to simulation settings.
- 3D deployment of UAVs is not considered in this study.

### 3.1. Proposed System Model

The cellular network’s users are randomly distributed in the ground network. In the traditional cellular networks, the base station (BS) provides communication convergence to the users. The BS coverage is fixed and limited for downlink and uplink data communication. The UAVs-BS solution provides cost-effective and mobility-oriented services. This type of solution is only available whenever there is a need to cover the area. If the network BS meets user needs, the UAVs-BS solution is not required. By adopting this strategy, the proposed solution provides a more effective, low-cost, and feasible solution. This study considers a downlink wireless system where the BS provides the services as usual, and the UAVs-BS is used for congested situations. This solution also addresses the overlap coverage situation.

The proposed solution is based on a statistically generic path loss model as used in [41]. The generic path loss model uses line-of-sight (LoS) and non-LoS links. To address the fading issue in the UAVs-BS, the proposed model uses Rician fading in LoS links, whereas the Rayleigh fading has been adopted for non-LoS links. The received signal stretch (RSS) from cellular users (CU) and the UAVs-BS is given as in Equations (1) and (2), respectively.

(1) $$TP_{LoS}\left(HD_{CU,UAVs-BS,}SD_{CU,UAVs-BS}\right)=TP_{CU,UAVs-BS}^{\varsigma \;}\mathrm{FLoS}$$

(2) $$TP_{LoS}\left(HD_{CU,UAVs-BS}SD_{CU,UAVs-BS}\right)=TP_{CU,UAVs-BS}^{\varsigma \;}\mathrm{Fnon}-\mathrm{LoS}$$

where the TP denotes the transmit power of the UAVs-BS, $HD_{CU,\;UAVs-BS}$ shows the horizontal distance of the CU and the UAVs-BS, and $SD_{CU,\;UAVs-BS}$ shows the spatial distance of the CU and the UAVs-BS. The FLoS and Fnon−LoS are the fading factors for the links. The probability of LoS connections between the CU and the UAVs-BS is presented in Equation (3).

(3) $$ProbLoS=\alpha (\frac{180}{\pi }.\arctan{\left(HD_{CU,UAVs-BS}\left(HD_{CU,UAVs-BS,}\right)-15\right)}^{\beta }$$

The α and β are constant values and change with dense and sparse cellular network conditions. The probability of non-LoS is 1-ProLoS. The average received power (RP) depends on the vertical and horizontal altitude. The RSS increases based on network and quality of services (QoS) requirements. With the increased altitude of the UAVs-BS, the coverage radius also increases, and when the altitude reaches a specific value, then the coverage radius decreases.

The UAVs-BS deployment’s main objective is to cover the maximum area. However, the overlap problem still exists, as well as the need for a demand coverage area. Finding a feasible altitude and providing maximum coverage is the main goal. The set of CUs is denoted as $Set_{CU}$ enclosed with the coverage area (CA), whereas the total coverage for the CU in the CA is shown in Equation (4).

(4) $$max\overset{L}{\underset{CU}{\sum }}|Set_{CU}|$$

The L shows the number of CAs, and the max shows the maximum altitude of the UAVs-BS. This shows that the number of CAs is not more than the number of UAVs-BS, and the HD considers the distance between the CU in the CA and the UAVs-BS. The proposed solution solves the optimization issue to maximize the CA of the CUs by proposing the altitude and coverage radius of the UAVs-BS.

### 3.2. Proposed Coverage Area Decision Model

The coverage ranges of the UAVs-BS for the CUs are based on data demand in the network. The proposed model uses the entropy method for optimization to measure the field distribution. The proposed model calculates the CA centre point by calculating the threshold of the potential value and distance. The threshold value is adopted from the [42]. According to this study, the area with a certain radius on a CA is considered an average CA. The proposed algorithm spots the CA of any size, achieves the maximum CA, and serves in a cellular network. After initializing the input, the next step is broadcasting the messages to call the drones, which have a central point CA. If drones get the minimum distance from the demand point, then they obtain the threshold value and end the session. The centre point determines and waits for a random time and repeats the process. All these steps in the CA decision process are shown in Algorithm 1.
**Algorithm 1: CA decision process**1.**Initializing**2.**Input:***CU, UAVs-BS, BS (a,b,c,d,e…..n), IDs & Location Information*3.**Output:** *Successful Coverage*4.***do*** Broadcast messages to call all the drone 5.obtain the CA center point6.***If*** min distance for demand point *then*
7.Obtain threshold value *else*8.***end if***9.Determine the CA centre point 10.Select CA ***else***11.*Wait for a random time and repeat the process*12.**end** if13.**end** process

### 3.3. Proposed UAVs-BS Mobility Model

After the CA initialization, the next step is the UAVs-BS mobility and selection of a selected CV for serving the cellular network. The UAVs-BS are working on the selected CA and enhancing the network performance. The selection of the UAVs-BS and its mobility in cellular networks is based on the link quality of the UAVs-BS and the CU density in the network. The proposed mobility model uses link quality and cellular network density because these parameters are more realistic in selecting the best UAVs-BS to serve the network. Sometimes, the cellular network is less congested, and the UAVs-BS mobility is based on coverage area decisions. The proposed mobility model improves the UAVs-BS mobility and only serves those areas which are more congested. The second parameters are link quality for better data communication with the ground BS and CUs. 

Most of the previous studies use the energy level or life of each UAV-BS because battery life is one of the significant parameters for decision making. However, the unpredictable cellular network condition is one of the causes of energy depletion. So, it is a better strategy to select the UAVs-BS by considering its link quality and user density at the ground. The proposed UAVs-BS mobility model decides the movement by using a decentralized decision-making method. This decision is also based on neighbour UAVs-BS within the CA radius range. Every UAV-BS is required to broadcast messages within a specific time for decision making. This model proposes maintaining the connectivity among neighbour UAVs, cellular networks, and the BS. The objective of the mobility model is to cover the CA, which is selected by using the proposed coverage area decision model. The UAVs-BS update their status by using control messages broadcasting where the message includes the current location of the UAVs-BS, the CA information, link quality, traffic density information, trailer, and addresses. The proposed mobility model has two main phases, including the information update phase and link quality plus a traffic density evaluation phase. The detail of these phases is discussed in the next sub-sections.

### 3.4. Information Update Phase

In this phase, the UAVs-BS update their location information and store it in their routing table. The table contains the UAVs-BS information by using the hop count between the UAVs-BS and the ground cellular BS. The neighbour UAVs-BS information is also updated with the ground BS and other neighbor UAVs. The link quality and traffic density information are also included in these control message beacons to update the network for decision making. All factors are combined, and the weightage factor is used for the decision to decide the UAVs-BS mobility in the selected CA. The computed weight decides the serving UAVs-BS for the cellular network. The UAVs work on demand to decide their availability to cover the cellular network. The UAVs-BS uses three types of packets: request packets (REQ-P), reply packets (REP-P), and decision packets (DEC-P).

**Figure 2:**
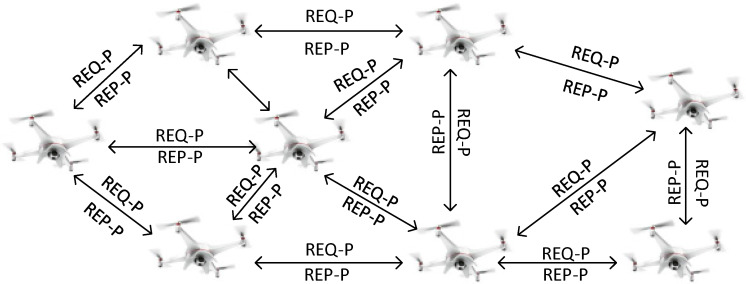
UAVs-BS status update process.

At the initial stage, the UAVs-BS sends the REQ-P message, which includes the UAV-ID, its own location information, CU density information received from cellular network BS, link quality of the source UAV, and route information. Every control message contains a timer and reset function. When the neighbour UAVs-BS receives the REQ-P, they generate the REP-P to confirm the neighbour UAV’s presence and location information. The control messages are around 12 bytes. The UAVs-BS maintain its record in the routing table. Figure 2 shows the UAVs-BS request and reply to messages exchange process.

### 3.5. Parameters Evaluation

**Figure 3:**
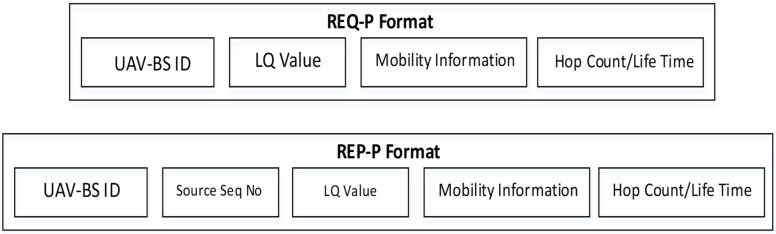
Request and reply packets format.

Link quality (LQ) is one of the significant factors for reliable data communication, high throughput, and other QoS operations. Due to the mobility of the UAVs-BS, the LQ is always on stack and unstable. This is the reason the proposed mobility model uses the LQ parameter by using the timer decision strategy as used in [43]. The timer decision is based on interval values 0 and 1 where the links are classified based on values. The LQ is evaluated by using the packet reception rate. There are three parameters to evaluate the LQ, including if the packet reception rate is between 10 to 90% considered connected, whereas a packet reception rate less than 10% means disconnected. Based on these parameters, the UAVs-BS defines its status with values 1 and 0. This information is stored in packet and traffic density information which is collected from the ground BS. After evaluating the LQ and traffic density, the weightage factor is applied for decision making and returns the value of 0 or 1. Figure 3 shows the packet format for the mobility model and the UAVs-BS selection. Algorithm 2 shows the UAVs-BS request and reply to messages process.
**Algorithm 2**: UAVs-BS request and reply to messages process1.**Initializing**2.**Input:***Source UAVs-BS IDs & Location Information*3.**Output:***UAVs-BS Placement Decision*4.***do*** Broadcast message to call all the UAVs-BS5.LQ+Traffic Density6.***If  ****LQ+Traffic Density==1, **then***7.*Select UAV-BS for placeman **else***12.Wait for a random time and repeat the process13.If REQ-P is received, **do**14.Data Forwarding **else**15.**end** if16.**end** while17.**end** process

The five levels of the proposed model are discussed in the above sub-sections. The coverage area decision model, the UAVs-BS mobility model, the information update phase, and the parameters evaluation model are designed to provide coverage facility to the cellular network by using UAV nodes that are controlled and manageable for reallocation.

## 4. Performance Analysis of the UAV-BS

The simulation is set to test the proposed solutions in terms of different performance parameters. The proposed solution is evaluated in the NS-2.34 simulator, which is integrated with SUMO and MOVE mobility modules. The NS-2.34 is based on the high enactment technical computing programming language employed for visualization, simulation, and plotting of mathematical data. UAV applications and their mobility patterns offer extensive feasible and cost-effective solutions and services. All the applications need stable routing without any delay and disconnection issues in the network. The UAV nodes need feasible data communication and security provision solutions with less overhead and fewer complexities. The cellular network nodes, the macrocell, and the UAV setting are set by using the editor. For UAV node mobility, the random walk mobility sets the altitude up to 200 to 500 feet. We considered some complex scenarios as well to test the proposed solution to check the UAV’s complex electromechanical systems in terms of delay and disconnection and set the parameters accordingly to avoid any delay or disconnection issues. The performance parameters to test the proposed solution are end-to-end delay, data throughput, and 0 network overhead. We also considered a delay factor before testing the proposed model and estimated the range of delay and disconnection. These factors help avoid instability in the network. The simulator uses two types of languages: C and object-oriented tool command language (OTcl). Table 2 shows the simulation parameters used to test the proposed solution.

### 4.1. Experiment Results of the Coverage Area Decision Model for the UAVs-BS

Coverage is always a challenging part of cellular networks. To address the coverage issue in cellular networks, unmanned aerial vehicles (UAVs) solutions have been adopted where the UAVs are working as a base station (BS). The proposed coverage area decision model for UAVs (CADM-UAVs) is evaluated in this section to check its performance to improve the ground network connectivity and provide high data rate services. The different simulation experiments are conducted to assess the performance of the proposed model. The proposed model is evaluated using network simulations for better coverage and decision making in terms of network delay, data throughput coverage, and spectral efficiency.

#### 4.1.1. Delay Analysis with Path Loss Exponent

**Figure 4:**
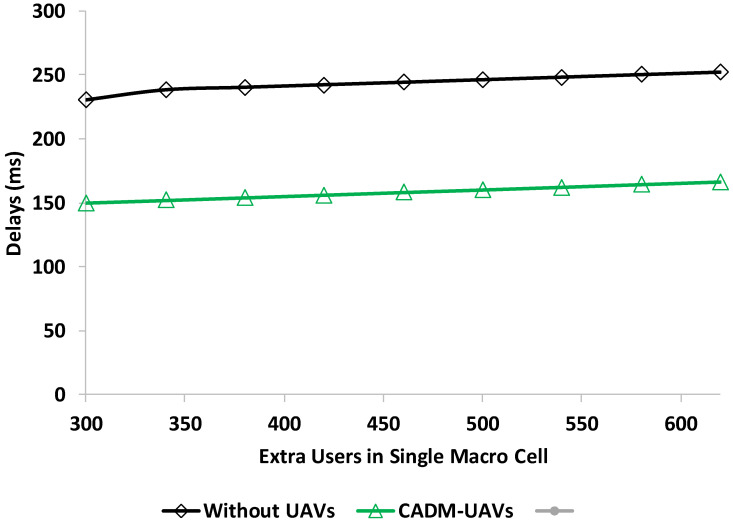
Delay analysis in the presence of user at cellular networks.

In the first experiment, the network delay with extra users in the cellular network is evaluated with or without UAV deployment. The UAV’s altitude is set at 200 to 500 feet because the high altitude provides less interference and a feasible line-of-sight (LoS). The delay threshold is set at 300 ms, whereas the distance of UAVs is set at 500 feet. Figure 4 shows the delay results in the presence of CADM-UAVs and without UAVs.

It is observed that the proposed CADM-UAVs have less delay compared to those without UAV deployment in cellular networks. When the users are 300 in a single macrocell, the delay of the proposed model is around 150, whereas the delay with a BS without UAVs is noted as 235 ms. The same trend increases when the users are around 600 in the single macrocell, where the proposed model achieved the result of 166 ms. The proposed CADM-UAVs achieve less delay and provide better coverage to the cellular network.

#### 4.1.2. Throughput Coverage Analysis with Path Loss Exponent and Users in Macro Cell

**Figure 5:**
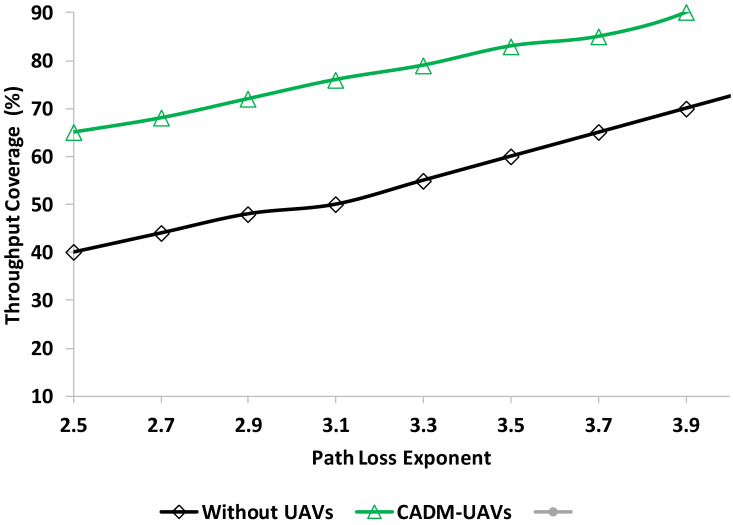
Throughput analysis in the presence of path loss exponent.

In this section, the second experiment is performed to check the performance of the proposed model CADM-UAVs compared to without UAV deployment to check the throughput coverage percentage. Figure 5 shows the throughput analysis.

**Figure 6:**
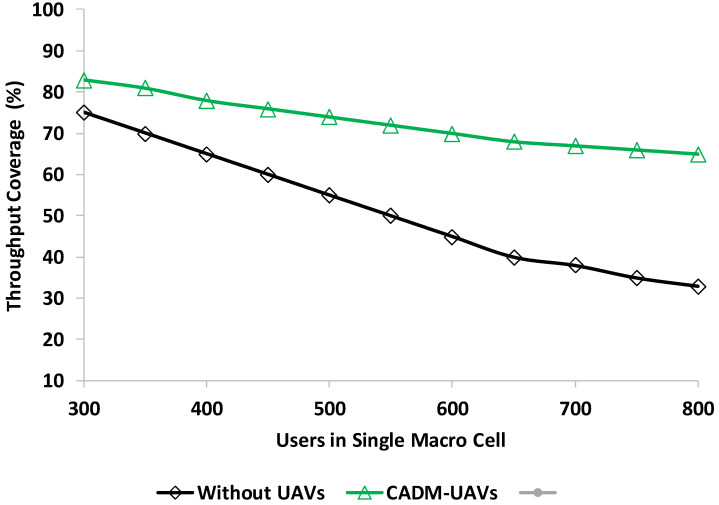
Throughput analysis in the presence of users in single macrocell.

In throughput analysis, the throughput coverage percentage is defined. The coverage percentage of users is defined with a high SINR from the threshold, which is around 65%. As shown in Figure 5 the proposed model throughput coverage is better compared with non-UAV networks. The path loss exponent refers to the distance between the transmitter and the receiver and is measured in meters. Figure 6 shows the throughput analysis in the presence of users in a single macrocell.

In this experiment, the throughput analysis is performed in the presence of several users in a single macrocell. Figure 6 indicates the better throughput percentage of the proposed model CADM-UAVs compared to the non-UAV network. This result is performed to set the threshold value, which is 65%. The placement of UAVs based on user demand improves the model performance, as depicted in the above experiment. It is indicated that when users are around 800 in a single macrocell, the performance of the proposed models is better, and at 70%, throughput converges. These results proved that the proposed CADM-UAVs model is one of the feasible solutions for cellular networks.

## 5. Conclusions

The paper proposed a cellular network coverage solution by using UAV nodes that are controlled and managed for reallocation and able to change position as per requirements. The UAVs have high line-of-sight (LoS), which makes these solutions more realistic for ground networks. The main objectives achieved in this paper are:

- Designed an optimal cellular coverage solution by establishing optimal numbers of BSs in the cellular network area and managing or reallocating the nodes as per requirements.
- The proposed solution offers optimal coverage area provision, especially in congested networks. Allocation of the UAVs-BS needs to be set with maximum and minimum vertical attitude.
- The proposed solutions are evaluated and tested in simulation in terms of data delay, data throughput, and network overhead.
- The proposed solution achieved better results in terms of placement in cellular networks.

The simulation results indicated the high performance of the proposed solution. In the future, the solution will be used in other scenarios such as underwater sensor networks (UWSN) and 6G high-speed networks. The UAV-based solution will cover more areas, especially in disaster situations, and facilitate the cellular network with more coverage. These solutions will help existing networks and provide cost-effective and feasible solutions for cover the network communication range. In future, we will deploy the proposed solution and integrate it with the 3D deployment strategy of UAVs to provide maximum wireless coverage for ground users.

## Figures and Tables

**Table 1:** Technical comparison of discussed studies.

S#	Ref.	Drone Placement		Drone Parameter			Multiple Drone	Placement Approach		Trust Mechanism
		Initial	Repositioning	User Density	Energy	Spectral Efficiency		Centralized	Distributed	
A Multitude of Done Base Stations										
1	3D Placement of Drone [24]	×	√	√	√	×	√	√	×	×
2	Dynamic Base Station Repositioning Model [26]	×	√	×	√	×	√	√	×	×
3	Emergency Ad Hoc Networks [8]	√	×	√	√	×	√	√	×	×
4	Backhaul-Aware Robust 3D Model [28]	×	×	√	×	×	√	√	×	×
5	User Association and Bandwidth Allocation Model [29]	×	√	×	×	√	√	√	×	×
6	IoT Connectivity in Radar Bands [30]	×	√	×	×	√	√	√	×	×
7	Aerial–Terrestrial Communications In [34],	√	×	×	×	√	√	√	×	×
8	UAV-Assisted Heterogeneous Model [35],	×	√	×	×	√	√	√	×	×
9	Solar Energy Harvesting [38]	√	×	×	×	√	√	√	×	×
10	SDN based manageable topology formation [39]	√	×	×	×	√	√	√	×	×
11	Leveraging Communicating UAVs [40]	×	√	√	×	√	√	√	×	×

**Table 2:** Simulation parameters.

S#	Parameters	Value
1	Network Size	4 × 4 Km
2	Time	900 s
3	Mobility Model	SUMO
4	No of UAVs	80
5	Drone Speed	0 to 60 Km/h
6	Communication rage	300 m for bases station and 1000 m for UAV/drone
7	Data size	1 to 1024 B
8	UAV Altitude	200–500 Feet
9	UAV Transmission Power	35 dBm
10	System Bandwidth	10 MHz
11	Active Users	400

## Data Availability

Not applicable.
